# GC/MS based metabolomics: development of a data mining system for metabolite identification by using soft independent modeling of class analogy (SIMCA)

**DOI:** 10.1186/1471-2105-12-131

**Published:** 2011-05-04

**Authors:** Hiroshi Tsugawa, Yuki Tsujimoto, Masanori Arita, Takeshi Bamba, Eiichiro Fukusaki

**Affiliations:** 1 Department of Bioengineering, Graduate School of Engineering, Osaka University, 2-1 Yamadaoka, Suita, Osaka 565-0871, Japan; 2 Department of Biophysics and Biochemistry, Graduate School of Science, The University of Tokyo, 7-3-1 Hongo, Bunkyo-ku, Tokyo 113-003, Japan

## Abstract

**Background:**

The goal of metabolomics analyses is a comprehensive and systematic understanding of all metabolites in biological samples. Many useful platforms have been developed to achieve this goal. Gas chromatography coupled to mass spectrometry (GC/MS) is a well-established analytical method in metabolomics study, and 200 to 500 peaks are routinely observed with one biological sample. However, only ~100 metabolites can be identified, and the remaining peaks are left as "unknowns".

**Result:**

We present an algorithm that acquires more extensive metabolite information. Pearson's product-moment correlation coefficient and the Soft Independent Modeling of Class Analogy (SIMCA) method were combined to automatically identify and annotate unknown peaks, which tend to be missed in routine studies that employ manual processing.

**Conclusions:**

Our data mining system can offer a wealth of metabolite information quickly and easily, and it provides new insights, particularly into food quality evaluation and prediction.

## Background

Metabolomics is based on biology, analytical chemistry, and information science, and it has become an important tool in many research areas [1-5]. The metabolome information can be used to extrapolate novel biological knowledge [1,6-8]. The main platforms in metabolomics studies are based on hybrid systems such as GC/MS, liquid chromatography (LC)/MS, and capillary electrophoresis (CE)/MS, all of which have been applied in many fields - including biomarker studies in medical diagnosis and quality evaluation and prediction in food science [2,3,5,9-11]. Among these platforms, GC/MS is a relatively mature method because the reproducible measurement is possible and many peaks (200 to 500) can be reliably obtained from a biological sample [1,3,12]. In addition, peak identification is straightforward when retention time (RT) and mass spectra data are compared to those of accumulated compound information in a laboratory (reference library). For these reasons, GC/MS is generally recognized as one of the most versatile and applicable platform in metabolomics.

Since GC/MS is mature enough to run a batch of analyses and to easily identify metabolite peaks, the development of a fast data analysis tool is essential [6,7]. Currently, peak identification and annotation is time-consuming when these processes are performed manually. Moreover, manual analysis results in serious problems in the accuracy of peak identification and annotation depending on the knowledge and expertise of individual researchers. Peak annotation is especially difficult because the extensive knowledge of fragmentation patterns by electron ionization (EI) is required. Therefore, it is an important challenge to develop data processing tools that identify and annotate metabolites easily, accurately, and rapidly.

Previous software platforms for peak identification utilize retention indexes that depend on an *n*-alkane mix (AMDIS [13], BinBase [14], MetaQuant [15], TagFinder [16], MetaboliteDetector [17]). But the retention index method requires some complicated procedures such as sample preparation and data analysis due to the *n*-alkane mix of the exogenous compounds. Moreover, the obtained metabolite information is limited to identifiable peaks because these platforms treat the ambiguous peak as "unknown". Therefore, many potentially interesting biomarkers tend to be disregarded.

There are several reasons why extracted peaks are left unidentified. First, peaks with a low signal-to-noise ratio, i.e., those with a large amount of noise, decrease the degree of coincidence (DOC) when compared to a reference library. Second, de-convolution may be unsuccessful because of co-elution (i.e., simultaneous elution of multiple compounds). Last and most importantly, no reference library is complete or covers information on all possible metabolites. If a certain metabolite is known to exist in a biological sample, a standard compound can be analyzed to resolve one unknown peak. However, if there is no information for a large number of unknown peaks, the cost of collecting standard compounds is prohibitively expensive; moreover, if a compound is not commercially available, the compound must be synthesized. For these reasons, it is important to deduce any kind of chemical information about unknown peaks.

We developed a data mining system to easily obtain metabolite information by using two mathematical methods. The first method is a Pearson's product-moment correlation coefficient for identification that we based on retention time and weighted mass spectrum [18,19]. Using 1) a retention time correction based on pseudo-internal standard and 2) a relaxed mass fitting to a reference library resulted in an identification process that was less dependent on column aging, column cuts, or column lot. In spectral comparison, higher masses are given more weight to reduce false positives and false negatives.

The second method is the Soft Independent Modeling of Class Analogy (SIMCA) [20] for the annotation of unknown peaks, and some techniques of SIMCA utilizing mass spectra have been developed, especially in toxic studies [21-25]. SIMCA is a supervised classification technique that is based on principal component analysis (PCA) [26], and it is useful for building multiple class models. New measurements are projected in each principle component (PC) space that describes a specific class, and the *F*-test is used to evaluate the Euclidean distances of the objects toward the model. We constructed the five chemical class models including amine, organic acid, fatty acid, sugar, and sugar phosphate groups as initiative. Using this method, we developed an annotation algorithm for unidentified peaks.

We utilized the free software MetAlign [27] for baseline correction, peak detection, and peak alignment. MetAlign has been a powerful tool for data preprocessing of GC/MS-based metabolomics [28,29]. The CSV format file exported from MetAlign can be analyzed by program written in Visual Basic, which software name is AIoutput. Our system and manual is given as additional files 1, 2, 3, and 4.

For validation, we performed two experiments. The first experiment included the standard mixtures: fifteen samples each mixed with 99 well-known standard compounds. In the standard-mix experiment, we demonstrated that the identification and annotation algorithms were robust and resulted in very few false positives or false negatives. The second experiment was a re-analysis of our published data. This experiment demonstrated that the required time for data processing was much shorter and that the novel system produced superior results. The proposed algorithm can be a powerful tool for quality evaluation and prediction, particularly in food science.

## Methods

### 1. Theoretical aspect

#### Retention time correction

Retention times provide important information for identifying metabolites. A common problem in accurate identification is chromatographic shift resulting from column aging or lot differences. To adjust such shifts, retention indexes based on an *n*-alkane mix are usually calculated. However, retention index correction has some disadvantages. First, the requirements for sample preparation, such as density adjustment between metabolites and an *n*-alkane mix, are complicated. Moreover, if the type or number of *n*-alkane mix used in each laboratory is different, results may not be compatible among laboratories. Therefore, we used stable metabolite peaks derived from biological samples as indexes in order to reduce the problem of chromatographic shift. Retention times from the reference library were updated by several pseudo-internal standards. The update method was as follows.

RT^new ^represents the retention time after update in the reference library, RT^old ^represents that of original data (See also additional file 4), rt^new ^and rt^old ^represent the retention time of the updated pseudo-internal standard and that of original one, respectively.

In an actual implementation, a user can choose up to eight compounds as pseudo-internal standards. The selection of standards is user-dependent, but the use of standards that result in early and late peaks is recommended for more accurate adjustment.

#### Peak identification

The most important information for peak identification is the mass spectrum of a compound. Pearson's product-moment correlation coefficient was used to measure the similarity of two mass spectra, which were represented as vectors of intensity for each integer mass unit. Because the EI ionization method is a hard ionization method, recorded mass spectra generally show larger intensities for lower masses than for higher masses. Because higher masses provide more reliable information for compound identification, higher masses were given larger weights in comparing two mass spectra. The identification method was as follows.

**E**_RT _and **L**_rt _represent the totally-weighted vectors of an extracted peak and of a reference compound, respectively. The parameter c presents the time width for a reference search. E^old ^and E^new ^represent the original intensity and the weighted intensity of the extracted spectrum, respectively. L^new ^and L^old ^represent the original intensity and the weighted intensity of a reference compound. For example, if an extracted peak, A, is eluted at 600 sec and the time width parameter c is set to 2 sec, the compounds from 598 to 602 sec in a reference library are selected as candidate matches. The compound from the reference library with the highest DOC when fitted to peak A is further selected as the match. If no candidate match is found, a prediction algorithm, described in the next section, is applied.

It should be noted that the time width was set by a user. Although pseudo-internal standard correction may impair accuracy compared to retention index correction, this relaxed mass fitting may have reduced the number of false negatives. This assertion is based on the assumption that mass spectra are more consistent and reliable than retention time for peak identification. In addition, although a few compounds have high similarity, the weighted mass spectra may have reduced false positives because the difference of the intensity in high masses was emphasized.

#### Peak prediction

SIMCA is a well-known pattern recognition method that distinguishes each class separately in a principal component (PC) space. SIMCA can also evaluate whether new objects belong to a specific model or not.

A training matrix, **X**, contains objects of different known classes. The sub-matrix, **X***_K_*, (*m *× *p*) contains *m *training objects belonging to class *K *that were measured at *p *variables. Each class training set is modeled separately by PCA. **X***_K _*is described with a score matrix, **T***_K_*, and loading matrix, **V***_K_*, as follows.

The number of important PCs, *r*, to describe the class, *K*, is usually determined by cross-validation [30,31]. **E***_K _*is the matrix containing the residuals. **X***_K _*is divided into two parts. One part  is described by *r *PCs, and the other **E***_K _*is the residuals of the PC space. The standard deviation of **E***_K_*, i.e., the residual standard deviation (RSD), and the RSD of new objects fitted to class *K *model are first compared, and then new objects are evaluated to determine whether they belong to class *K*. The RSD of **E***_K _*is, in fact, a measure for the Euclidean distance of the class *K *objects toward the *r *PC space.

 represents the residual of object, *k*, of the class *K *training set at variable *i*.

To predict whether an object, , belongs to the class *K*, it is projected on the space defined by the selected PCs of the class *K *training set.

 represents the predicted object, , in the space of the class *K *training set. The residual vector  of object  is calculated as follows.

And the RSD, *s_j _*i.e., a Euclidean distance taking into account the degree of freedom, is obtained as follows.

One determines whether the residual variances  and  are significantly different by calculating the *F*-value compared to the tabulated critical *F-*crit for (*m *- *r *-1) and (*m *- *r *-1)^2 ^degree of freedom.

If the residual variances  and  are significantly different, the new object will not be classified into the class *K*. On the other hand, if the residual variances are not significantly different, the new object will be classified into class *K*. The test is performed under all classes.

In the AIoutput software, SIMCA is applied to unidentified peaks to classify them into a metabolite group (sugar, sugar phosphate, organic acid, amine, or fatty acid). If an unidentified peak could be classified into multiple groups, the group associated with the largest *p*-value is chosen. In this study, however, unknown peaks were rarely classified into multiple groups (3 out of 84 cases in re-analysis). If an unidentified peak is not classified into any class, the peak is ultimately reported as unknown. But the AIoutput software creates an organized data matrix that includes the unknown peak information. This type of output represents the ultimate goal of metabolomics studies, which is a comprehensive analysis of all metabolites in the biological samples.

### 2. Practical workflow

#### Construction of the SIMCA model

We prepared five metabolite groups for annotation: sugar, sugar phosphate, organic acid, fatty acid, and amine, and 12, 10, 12, 9, and 13 compounds, respectively, were prepared for the training matrix (Table 1). We used the relative intensities of each mass value ranging *m/z *85 to 500 as variables in the SIMCA model.

**Table 1:** Compounds used in the training set for the SIMCA method

Class	Name	IUPAC	CAS	KEGG
Sugar	Fructose	(3S,4R,5R)-2-(hydroxymethyl)oxane-2,3,4,5-tetrol	57-48-7	C00095
	Galactose	(3R,4S,5R,6R)-6-(hydroxymethyl)oxane-2,3,4,5-tetrol	59-23-4	C00124
	Glucose	(3R,4S,5S,6R)-6-(hydroxymethyl)oxane-2,3,4,5-tetrol	50-99-7	C00031
	Glycerol	propane-1,2,3-triol	56-81-5	C00116
	Maltose	(2R,3S,4S,5R,6R)-2-(hydroxymethyl)-6-[(2R,3S,4R,5R)-4,5,6-trih ydroxy-2-(hydroxymethyl)oxan-3-yl]oxyoxane-3,4,5-triol	69-79-4	C00208
	Sucrose	(2R,3R,4S,5S,6R)-2-[(2S,3S,4S,5R)-3,4-dihydroxy-2,5-bis(hydrox ymethyl)oxolan-2-yl]oxy-6-(hydroxymethyl)oxane-3,4,5-triol	57-50-1	C00089
	Trehalose	(2R,3S,4S,5R,6R)-2-(hydroxymethyl)-6-[(2R,3R,4S,5S,6R)-3,4,5-t rihydroxy-6-(hydroxymethyl)oxan-2-yl]oxyoxane-3,4,5-triol	99-20-7	C01083
	Xylitol	(2R,4S)-pentane-1,2,3,4,5-pentol	83-99-0	C00379
	Inositol	cyclohexane-1,2,3,4,5,6-hexol	87-89-8	C00137
	Sorbitol	(2R,3R,4R,5S)-hexane-1,2,3,4,5,6-hexol	50-70-4	C00794
	Ribose	(3R,4S,5R)-5-(hydroxymethyl)oxolane-2,3,4-triol	50-69-1	C00121
	Maltitol	(2S,3R,4R,5R)-4-[(2R,3R,4S,5S,6R)-3,4,5-trihydroxy-6-(hydroxyl methyl)oxan-2-yl]oxyhexane-1,2,3,5,6-pentol	81025-03-8	C13542

Sugar phosphate	Fructose-6-phosphate	[(2R,3R,4S)-2,3,4,6-tetrahydroxy-5-oxohexyl] dihydrogen phosphate	643-13-0	C00085
	Glucosamine-6-phosphate	[(2R,3S,4R,5R)-5-amino-2,3,4-trihydroxy-6-oxohexyl] dihydrogen phosphate	3616-42-0	C00352
	Glycerol-2-phosphate	1,3-dihydroxypropan-2-yl phosphate	17181-54-3	C02979
	Arabinose-5-phosphate	[(2R,3R,4S)-2,3,4-trihydroxy-5-oxopentyl] phosphate	13137-52-5	C01112
	Ribulose-5-phosphate	[(2R,3R)-2,3,5-trihydroxy-4-oxopentyl] phosphate	551-85-9	C00199
	Sorbitol-6-phosphate	2,3,4,5,6-pentahydroxyhexyl phosphate	20479-58-7	C01096
	Phosphoenolpyruvic acid	2-phosphonooxyprop-2-enoic acid	138-08-9	C00074
	Deoxyribose-5'-phosphate	[(2R,3S)-3-hydroxyoxolan-2-yl]methyl hydrogenphosphate	7685-50-9	C00673
	Glucose-6-phosphate	[(2R,3S,4S,5R)-3,4,5,6-tetrahydroxyoxan-2-yl]methyl dihydrogen phosphate	56-73-5	C00092
	Ribulose-1,5-bisphosphate	(2,3-dihydroxy-4-oxo-5-phosphonatooxypentyl)	24218-00-6	C01182

Organic acid	Oxalic acid	oxalic acid	144-62-7	C00209
	Isocitric acid	1-hydroxypropane-1,2,3-tricarboxylic acid	320-77-4	C00311
	2-Isopropylmalic acid	2-hydroxy-2-propan-2-ylbutanedioic acid	3237-44-3	C02504
	Succinic acid	butanedioic acid	110-15-6	C00042
	Maleic acid	(Z)-but-2-enedioic acid	110-16-7	C01384
	Malic acid	2-hydroxybutanedioic acid	617-48-1	C00711
	Malonic acid	propanedioic acid	141-82-2	C00383
	Glutaric acid	pentanedioic acid	110-94-1	C00489
	Glycolic acid	2-hydroxyacetic acid	79-14-1	C00160
	Citramalic acid	2-hydroxy-2-methylbutanedioic acid	2306-22-1	C00815
	Citric acid	2-hydroxypropane-1,2,3-tricarboxylic acid	77-92-9	C00158
	Methylmalonic acid	2-methylpropanedioic acid	516-05-2	C02170

Fatty acid	Elaidic acid	(E)-octadec-9-enoic acid	112-79-8	C01712
	Heptadecanoic acid	heptadecanoic acid	506-12-7	Not found
	Icosanoic acid	icosanoic acid	506-30-9	C06425
	Lauric acid	dodecanoic acid	143-07-7	C02679
	Lignoceric acid	tetracosanoic acid	557-59-5	C08320
	n-Caprylic acid	octanoic acid	124-07-2	C06423
	Nonanoic acid	nonanoic acid	112-05-0	C01601
	Octacosanoic acid	octacosanoic acid	506-48-9	Not found
	Palmitoleic acid	(E)-hexadec-9-enoic acid	373-49-9	C08362

Amine	Dopamine	4-(2-aminoethyl)benzene-1,2-diol	51-61-6	C03758
	Cadaverine	pentane-1,5-diamine	462-94-2	C01672
	n-Butylamine	butan-1-amine	109-73-9	C18706
	Putrescine	butane-1,4-diamine	110-60-1	C00134
	Tyramine	4-(2-aminoethyl)phenol	51-67-2	C00483
	Isobutylamine	2-methylpropan-1-amine	78-81-9	C02787
	2-Aminoethanol	2-aminoethanol	141-43-5	C00189
	1,3-Propanediamine	N',N'-dimethylpropane-1,3-diamine	109-76-2	C00986
	n-Propylamine	propan-1-amine	107-10-8	Not found
	Tryptamine	2-(1H-indol-3-yl)ethanamine	61-54-1	C00398
	Histamine	2-(1H-imidazol-5-yl)ethanamine	51-45-6	C00388
	1-Methylhistamine	2-(1-methylimidazol-4-yl)ethanamine	501-75-7	C05127
	Serotonin	3-(2-aminoethyl)-1H-indol-5-ol	50-67-9	C00780

Compounds in each metabolite group were randomly selected from our reference library based on the metabolite feature. The popular name, IUPAC name, CAS registry number, and KEGG ID were described, respectively.

#### Standard mixture experiment

In order to validate the accuracy of our identification and annotation algorithms, we performed the following verification experiment. Standard compounds (99 total, see Table 2 and 3) were dispensed into 2 ml eppendorf tubes at three concentrations (5 μl, 10 μl, or 15 μl each standard solution of 10 mM). For each pattern, five tubes were prepared (15 standard mixtures in total). Any methanol in the mixtures was evaporated in a vacuum centrifuge dryer for 1 hour, and the mixtures were freeze-dried overnight.

**Table 2:** 43 out of 99 compounds included in the five classes

Class	Name	IUPAC	Predicted Name
Organic acid	Citramalic acid	2-hydroxy-2-methylbutanedioic acid	Organic acid
	Citric acid	2-hydroxypropane-1,2,3-tricarboxylic acid	Organic acid
	Fumaric acid	(*E*)-but-2-enedioic acid	Organic acid
	Glycolic acid	2-hydroxyacetic acid	Organic acid* and Sugar
	Maleic acid	(*Z*)-but-2-enedioic acid	Organic acid
	Malic acid	2-hydroxybutanedioic acid	Organic acid
	Malonic acid	propanedioic acid	Organic acid
	Mandelic acid	2-hydroxy-2-phenylacetic acid	Organic acid
	Oxalic acid	oxalic acid	Organic acid
	Oxamic acid	oxamic acid	Organic acid
	Shikimic acid	(3*R*,4*S*,5*R*)-3,4,5-trihydroxycyclohexene-1-carboxylic acid	No annotation
	Succinic acid	butanedioic acid	Organic acid

Sugar	Arabinose	(2*S*,3*R*,4*R*)-2,3,4,5-tetrahydroxypentanal	Sugar
	Arabitol	(2*R*,4*R*)-pentane-1,2,3,4,5-pentol	Sugar
	Fructose	(3*S*,4*R*,5*R*)-2-(hydroxymethyl)oxane-2,3,4,5-tetrol	Sugar
	Galactose	(3*R*,4*S*,5*R*,6*R*)-6-(hydroxymethyl)oxane-2,3,4,5-tetrol	Sugar
	Glucose	(3*R*,4*S*,5*S*,6*R*)-6-(hydroxymethyl)oxane-2,3,4,5-tetrol	Sugar
	Inositol	cyclohexane-1,2,3,4,5,6-hexol	Sugar* and Organic acid
	Maltose	(2*R*,3*S*,4*S*,5*R*,6*R*)-2-(hydroxymethyl)-6-[(2*R*,3*S*,4*R*,5*R*)-4,5,6-trihydrox y-2-(hydroxymethyl)oxan-3-yl]oxyoxane-3,4,5-triol	Sugar
	Mannose	(3*S*,4*S*,5*S*,6*R*)-6-(hydroxymethyl)oxane-2,3,4,5-tetrol (2*R*,3*R*,4*S*,5*S*,6*R*)-2-[(2*S*,3*S*,4*R*,5*R*)-4-hydroxy-2,5-bis(hydroxymethyl)	Sugar
	Melezitose	-2-[(2*R*,3*R*,4*S*,5*S*,6*R*)-3,4,5-trihydroxy-6-(hydroxymethyl)oxan-2-yl]ox yoxolan-3-yl]oxy-6-(hydroxymethyl)oxane-3,4,5-triol	Sugar
	Ribitol	pentane-1,2,3,4,5-pentol	Sugar
	Ribose	(3*R*,4*S*,5*R*)-5-(hydroxymethyl)oxolane-2,3,4-triol	Sugar
	Sucrose	(2*R*,3*R*,4*S*,5*S*,6*R*)-2-[(2*S*,3*S*,4*S*,5*R*)-3,4-dihydroxy-2,5-bis(hydroxymet hyl)oxolan-2-yl]oxy-6-(hydroxymethyl)oxane-3,4,5-triol	Sugar
	Threitol	(2*R*,3*R*)-butane-1,2,3,4-tetrol	Sugar
	Trehalose	(2*R*,3*S*,4*S*,5*R*,6*R*)-2-(hydroxymethyl)-6-[(2*R*,3*R*,4*S*,5*S*,6*R*)-3,4,5-trihyd roxy-6-(hydroxymethyl)oxan-2-yl]oxyoxane-3,4,5-triol	Sugar
	Xylose	(2*S*,3*R*,4*S*,5*R*)-oxane-2,3,4,5-tetrol	Sugar
	Glycerol	propane-1,2,3-triol	Sugar

Sugar phosphate	Ribulose-5-phosphate	[(2*R*,3*R*)-2,3,5-trihydroxy-4-oxopentyl] dihydrogen phosphate	Sugar phosphate

Amine	Cadaverine	pentane-1,5-diamine	Amine
	Dopamine	4-(2-aminoethyl)benzene-1,2-diol	Amine
	Isobutylamine	2-methylpropan-1-amine	Amine
	*n*-Butylamine	butan-1-amine	Amine
	*n*-Propylamine	propan-1-amine	Amine
	Putrescine	butane-1,4-diamine	Amine
	Spermidine	*N'*-(3-aminopropyl)butane-1,4-diamine	No annotation
	Spermine	*N,N'*-bis(3-aminopropyl)butane-1,4-diamine	No annotation
	Tyramine	4-(2-aminoethyl)phenol	Amine
	Histamine	2-(1H-imidazol-5-yl)ethanamine	Amine
	Serotonin	3-(2-aminoethyl)-1H-indol-5-ol	Amine
	Tryptamine	2-(1H-indol-3-yl)ethanamine	Amine

Fatty acid	Heptadecanoic acid	heptadecanoic acid	Fatty acid
	Octadecanoic acid	octadecanoic acid	Fatty acid

Table 2 shows 43 standard compounds classified to the five metabolite groups constituting the SIMCA method. Table 3 shows the remaining 56 standard compounds. Table 2 and 3 also show the predicted name of each compound by the SIMCA algorithm. If a compound was classified into some groups, the groups were fastened by "and". The asterisk (*) indicates the group with higher *p*-value. If a compound was not classified into any groups, the predicted name was described as "No annotation".

**Table 3:** 56 out of 99 compounds not included in the five classes

Class	Name	IUPAC	Predicted Name
Benzene	4-Aminobenzoic acid	4-aminobenzoic acid	No annotation
	Benzoic acid	benzoic acid	No annotation
	*o*-Toluic acid	2-methylbenzoate	No annotation
	Phenylalanine	(2*S*)-2-amino-3-phenylpropanoic acid	No annotation
	Tyrosine	(2*S*)-2-amino-3-(4-hydroxyphenyl)propanoic acid	No annotation
	Ferulic acid	(*E*)-3-(4-hydroxy-3-methoxyphenyl)prop-2-enoic acid	No annotation
	Dopa	(2*S*)-2-amino-3-(3,4-dihydroxyphenyl)propanoic acid	No annotation

Alpha-Keto acid	2-Oxoglutaric acid	2-oxopentanedioic acid	No annotation
	Pyruvic acid	2-oxopropanoic acid	Amine

Indole, Imidazole	Histidine	(2*S*)-2-amino-3-(1H-imidazol-5-yl)propanoic acid	No annotation
	Histidinol	2-amino-3-(1H-imidazol-5-yl)propan-1-ol	No annotation
	Tryptophan	(2*S*)-2-amino-3-(1H-indol-3-yl)propanoic acid	No annotation

Purine, Pyrimidine	Adenine	7H-purin-6-amine	No annotation
	Caffeine	1,3,7-trimethylpurine-2,6-dione	No annotation
	Cytosine	6-amino-1H-pyrimidin-2-one	No annotation
	Guanine	2-amino-3,7-dihydropurin-6-one	No annotation
	Inosine	9-[(2*R*,3*R*,4*S*,5*R*)-3,4-dihydroxy-5-(hydroxymethyl)oxolan-2-yl]-3H-p urin-6-one	No annotation
	Thymine	5-methyl-1H-pyrimidine-2,4-dione	No annotation
	Uracil	1H-pyrimidine-2,4-dione	No annotation
	Xanthine	3,7-dihydropurine-2,6-dione	No annotation

Amino acid	2-Aminobutyric acid	2-aminobutanoic acid	No annotation
	2-Aminoisobutyric acid	2-amino-2-methylpropanoic acid	No annotation
	4-Aminobutyric acid	4-aminobutanoic acid	Amine
	Alanine	(2*S*)-2-aminopropanoic acid	No annotation
	Allothreonine	(2*S*,3*S*)-2-amino-3-hydroxybutanoic acid	No annotation
	Asparagine	(2*S*)-2,4-diamino-4-oxobutanoic acid	No annotation
	Aspartic acid	(2*S*)-2-aminobutanedioic acid	No annotation
	Citrulline	(2*S*)-2-amino-5-(carbamoylamino)pentanoic acid	No annotation
	Cysteine	(2*R*)-2-amino-3-sulfanylpropanoic acid	No annotation
	Glutamic acid	(2*S*)-2-aminopentanedioic acid	No annotation
	Glutamine	(2*S*)-2,5-diamino-5-oxopentanoic acid	No annotation
	Glycine	2-aminoacetic acid	Amine
	Glycyl-glycine	2-[(2-aminoacetyl)amino]acetic acid	No annotation
	Homoserine	2-amino-4-hydroxybutanoic acid	No annotation
	Isoleucine	(2*S*,3*S*)-2-amino-3-methylpentanoic acid	No annotation
	Leucine	(2*S*)-2-amino-4-methylpentanoic acid	No annotation
	Lysine	(2*S*)-2,6-diaminohexanoic acid	No annotation
	Methionine	(2*S*)-2-amino-4-methylsulfanylbutanoic acid	No annotation
	*N*-Acetyl-DL-valine	2-acetamido-3-methylbutanoic acid	No annotation
	Ornithine	(2*S*)-2,5-diaminopentanoic acid	No annotation
	Proline	(2*S*)-pyrrolidine-2-carboxylic acid	No annotation
	Sarcosine	2-(methylamino)acetic acid	No annotation
	Serine	(2*S*)-2-amino-3-hydroxypropanoic acid	No annotation
	Threonine	(2*S*,3*R*)-2-amino-3-hydroxybutanoic acid	No annotation
	Valine	(2*S*)-2-amino-3-methylbutanoic acid	No annotation
	*β*-Alanine	3-aminopropanoic acid	No annotation

Other	2-Hydroxypyridine	1H-pyridin-2-one	No annotation
	4-Hydroxypyridine	1H-pyridin-4-one	No annotation
	Phosphoric acid	phosphate	Sugar phosphate
	Kojic acid	5-hydroxy-2-(hydroxymethyl)pyran-4-one	No annotation
	Nicotinic acid	pyridine-3-carboxylic acid	No annotation
	Quinic acid	(3*R*,5*R*)-1,3,4,5-tetrahydroxycyclohexane-1-carboxylic acid	No annotation
	Propyleneglycol	propane-1,2-diol	No annotation
	Creatinine	2-amino-3-methyl-4H-imidazol-5-one	No annotation
	Urea	urea	Organic acid
	Ascorbic acid	(2R)-2-[(1S)-1,2-dihydroxyethyl]-4,5-dihydroxyfuran-3-one	No annotation

The detail is shown in Table 2.

Sample derivatization procedures were followed previously [5]. In brief, methoxyamine hydrochloride in pyridine was added for oximation, and *N*-methyl-*N*-(trimethylsilyl) trifluoroacetamide (MSTFA) was added for silylation, and 1 μl of each mixture was injected in the split mode (25:1, v/v). Auto-sampler was a 7683B series injector (Agilent Co., Palo Alto, CA), and gas chromatograph was a 6890N (Agilent Co., Palo Alto, CA), and mass spectrometer was a Pegasus III TOF (LECO, St. Joseph, MI). The column was a 30 m × 0.25 mm i.d. fused silica capillary column coated with 0.25 μm CP-SIL 8 CB low bleed/MS (Varian Inc., Palo Alto, CA). The front inlet temperature was 230°C. The helium gas flow rate through the column was 1 ml/min. The column temperature was held at 80°C for 2 min isothermally and then was raised by 15°;C/min to 330°C and was held there for 6 min isothermally. The transfer line and ion source temperatures were 250°C and 200°C, respectively. 20 scans per second were recorded over the mass range 85-500 *m/z*.

MS data were exported in the netCDF format (See additional file 5). Fifteen chromatograms were peak-detected and aligned using the MetAlign software (Wageningen UR, The Netherlands, freely available at http://www.pri.wur.nl/UK/products/MetAlign/). The resulting data was exported in the CSV-format file (See additional file 6). After updating retention times of our reference library by the pseudo-internal standard correction method (see above), peak identification and annotation were executed in the AIoutput software.

#### Published data experiment

In order to verify the utility of our system, we re-analyzed data from our previous work that is reported in Pongsuwan W *et al. *[5]. The analytical method used for this experiment was exactly the same as that used for the standard mixture experiment.

## Result and Discussion

### Validation and optimization of the SIMCA model

**Figure 1:**
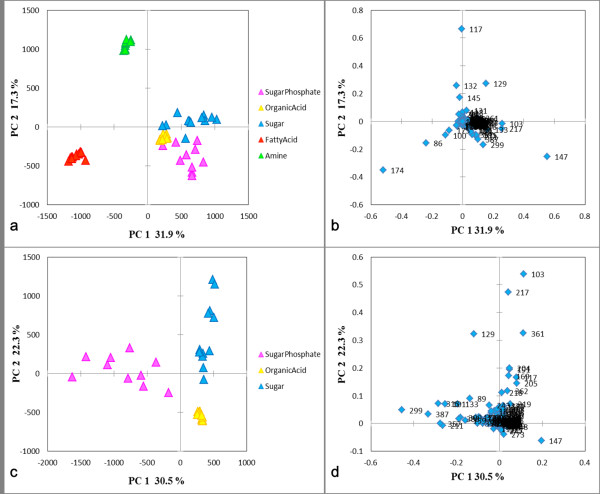
**Model evaluation**. (a), (b) The PCA score and loading plot including all compound groups. (c), (d) The score and loading plot including organic acid, sugar, and sugar phosphate groups. Mean centering was used in the data preprocessing. The legend shows each metabolite group. X-axis and Y-axis describe the first and second PCs, respectively.

It was important to evaluate independence of five class models. We performed PCA toward the data matrix (56 × 416), i.e., spectral vectors of 56 compounds used in the SIMCA model (Figure 1a and 1b). The metabolite groups were clearly separated by the first and second PCs, and the amine and fatty acid groups were especially independent. As shown in Figure 1b, the loading plot shows that the *m/z *86 and 174 contributed to the discrimination of amine group, and the *m/z *117, 129, and 132 contributed to the discrimination of fatty acid group. To investigate the features of organic acid, sugar, and sugar phosphate groups in detail, we applied PCA to the data matrix (34 × 416) including only the three groups. As shown in Figure 1c and 1d, the *m/z *299 clearly discriminated the sugar phosphate group, and the *m/z *147 was a characteristic mass to the organic acid group.

After we applied PCA to five metabolite groups individually, we optimized each model using interclass distance as follows.

*s*_12 _denotes the interclass residual when Class 1 objects were projected into the PC space of Class 2. *r*_2 _and *m*_1 _represent the factor number of Class 2 and the number of training objects for Class 1, respectively. It should be noted that the interclass residual of Class 1 described by Class 2 space was different from that of Class 2 described by Class 1 space (*s*_12 _≠ *s*_21_). For this reason, we used an interclass distance *D*_12 _as the distance between class models, and the values larger than one indicate real differences [20]. Higher distances indicate that models are more independent of one another. If two models are not independent, the interclass distance is close to zero. Table 4 shows the interclass distance, PC number, and the important *m/z *used in the SIMCA model. The classes were largely independent of one another. In addition, because only one PC was used as the latent variable for all metabolite groups, the model should be robust and less over-fitted. In the cross validation, the misclassifications were nothing (Table 5). This result shows that a good model can be constructed for annotating metabolites from mass spectra.

**Table 4:** Interclass distance resulting from SIMCA

Class name	Sugar phosphate	Organic acid	Sugar	Amine	Fatty acid	PC number	Important *m/z*
Sugar phosphate	0.00	1.21	1.05	1.85	1.79	1	89, 147, 217, **299**
Organic acid	1.21	0.00	1.46	3.81	4.38	1	101, 133, **147**
Sugar	1.05	1.46	0.00	2.72	2.53	1	89, **103**, 147, 217
Amine	1.85	3.81	2.72	0.00	4.32	1	86, 100, **174**
Fatty acid	1.79	4.38	2.53	4.32	0.00	1	**117**, 129, 132, 145

We used only one PC for all groups in order to make a robust model without over-fit. A distance close to zero indicates that the two classes are virtually identical, and the value above 1.0 indicates real differences. The important *m/z *contributed to a model was indicated, and the most important *m/z *was shown by bold type.

**Table 5:** Cross validation of SIMCA model

Actuals\Prediction	phosphate Sugar	Organic acid	Sugar	Amine	Fatty acid
Sugar phosphate	10	0	0	0	0
Organic acid	0	12	0	0	0
Sugar	0	0	12	0	0
Amine	0	0	0	13	0
Fatty acid	0	0	0	0	9

Cross validation was automatically performed by Pirouetto 4.0 software (InfoMetrix).

### Identification and annotation accuracies by the standard-mix experiment

Table 6 shows the result of peak identification by Manual, ChromaTOF software, and the AIoutput software, respectively. Our system required only two minutes for analyzing the CSV-format file, and all 99 compounds in 15 samples were unmistakably identified. Several amino acids generate two peaks due to different degrees of silylation at primary amines, and sugars generate several peaks due to their geometric isomers derived from in the oxime reaction [32-34]. Such peaks were also identified accurately. Although there were the ten false positives, some of these false positive might have been generated by additional reactions in the derivatization process and by the pyrolysis reaction in the front inlet and capillary column [33,34]. The formation of TMS-pyroglutamate from TMS-glutamate is a characteristic example of an additional reaction in the derivatization process [34]. Moreover, we also confirmed the accuracy of annotation algorithm (see Table 2 and 3). Some compounds of organic acid and sugar groups were classified into two groups. Although the organic acid and sugar groups were relatively similar as shown in Figure 1 and Table 4, the end result by *p*-value was correct. Some compounds including an amino functional group were classified to amine group. Despite some misclassifications, however, the result suggests that our annotation algorism is acceptable because the mass fragmentation is not always dependent to the functional groups. In the fragmentation pattern, pyruvic acid, phosphoric acid, and urea have *m/z *174, *m/z *299, and *m/z *147 as high intensity mass, respectively. Spermidine and spermine have the unique mass fragmentation patterns different in amine group (See additional file 2).

**Table 6:** Peak identification results by manual, ChromaTOF and the AIoutput software

	Analysis time	False negatives	False positives
Manual	39 ± 15 h	12 ± 6	5 ± 2
ChromaTOF	20 sec	70	5
AIoutput	2 min	0	10

Manual analysis was performed by six skilled people in our laboratory. ChromaTOF software identified the compounds based on the NIST library. The AIoutput software identified compounds based on our reference library.

### System evaluation by the data re-analysis

**Figure 2:**
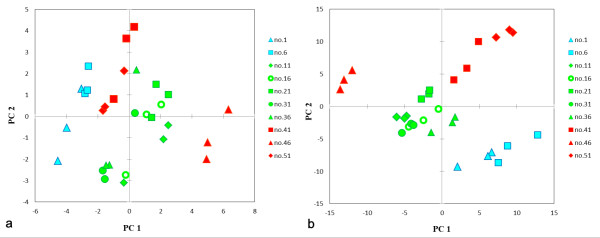
**Result comparison**. (a) The PCA score plot made by our previous method. (b) The PCA score plot made by our new system. The legend shows the ranking of the Japanese green tea samples. The variations in each group were relatively small, and each tea grade was clearly better separated in the second PC with the new system.

We re-analyzed the published data in order to show the utility of our system. The biological samples used were Japanese green teas that had been ranked in an agricultural fair [5]. Our system recognized 231 peaks in these chromatograms, and offered an organized data matrix without any missing values (See additional file 7). Out of 231 peaks, 112 were matched with compounds from our reference library, and 83 peaks were classified into a predicted metabolite groups; organic acid, sugar, sugar phosphate, amine, and fatty acid groups included 56, 18, 3, 6 and 0 peaks, respectively. We applied the organized data matrix to PCA (Figure 2). Figure 2a and 2b represent the PCA score plots from the data matrix obtained by the previous analysis [5] and the new analysis, respectively. Our new system produced better classification, and the second PC space closely correlated with tea grades. Moreover, the required time for data processing was about 30 min.

**Figure 3:**
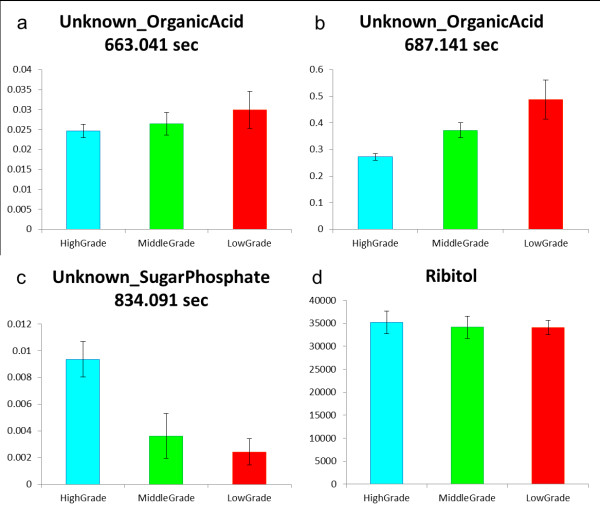
**Annotated peaks information**. (a), (b), (c) The peak height of three important metabolites for describing the tea grade in the second PC space. (d) The peak height of ribitol. The peaks of the annotated metabolites were scaled relative to the ribitol peak. The graph title indicates their annotated names and their respective retention times. These three peaks clearly varied with tea quality.

**Figure 4:**
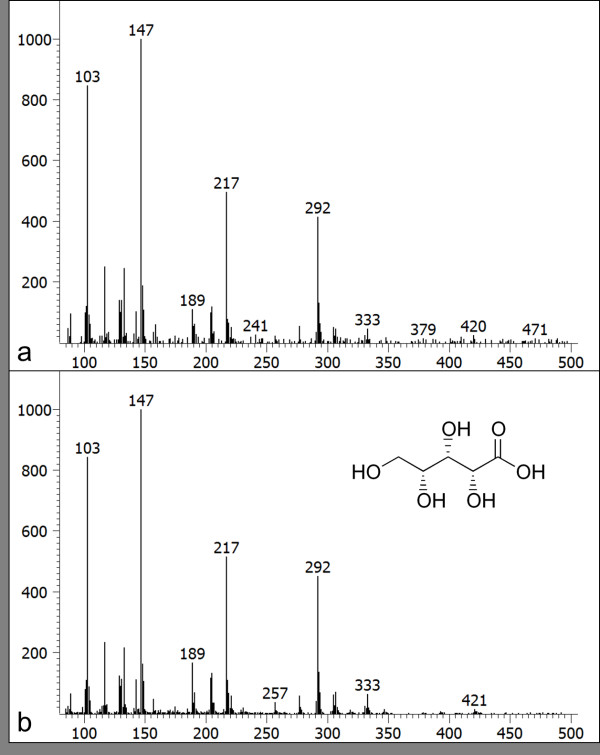
**Xylonic acid mass spectra**. (a) Mass spectra of an annotated metabolite in a Japanese green tea sample. This metabolite is the same as Fig. 3a. (b) Xylonic acid mass spectra.

Because the second PC correlated with tea quality, we examined the loading of the second PC (data not shown). In addition to some identified metabolites, two annotated metabolites (Figure 3a and 3b) positively contributed to the second PC, and one annotated metabolite (Figure 3c) contributed negatively (we also confirmed the mass spectra of these annotated peaks by manual). The amounts of three metabolites clearly differed among tea grades. Note here that the second PC was insensitive to the analytical order because the tea samples had been randomly analyzed by GC-TOF/MS, also note that ribitol could be reliably used as the internal standard (Figure 3d). Of these three annotated peaks, we identified one metabolite as xylonic acid by our additional investigation (Figure 4). Xylonic acid is a minor sugar acid, and this is new insight into Japanese green tea. We also examined standard compounds of xylitol and xylose in order to confirm whether xylonic acid was generated from these compounds because of additional reaction in the derivatization process (data not shown).

## Conclusion

The purpose of metabolomics is a comprehensive analysis of metabolites in biological samples. GC-TOF/MS offers highly reproducible information on primary metabolites. Our new data analysis tool provided the useful metabolite information and the organized data matrix accurately and rapidly. The system identified compounds by a retention time correction based on pseudo-internal standard and a relaxed mass fitting without requiring complicated sample preparation procedures, such as density control. This system can be also used to re-analyze past data if the reference library is provided. As shown by the re-analysis of our published data, novel knowledge about Japanese green tea research is available for quality evaluation and prediction in food science. Our study suggests that researchers can achieve high-quality GC/MS-based metabolomics relatively easily. However, GC-TOF/MS is comparatively expensive; therefore, we are working to develop a similar system for GC-Q/MS, which is considerably less expensive. Moreover, this method will be also used to develop the "Known" and "Known unknown" metabolite library database for non-targeted metabolomics analysis.

## Authors' contributions

EF initiated and supervised the project. HT improved the initial concept and designed and implemented the program, and wrote the manuscript. YT and HT prepared the reference library of 500 compounds. MA contributed to the system performance and also contributed to writing the paper. EF and TB proposed the retention time correction method. EF and TB also contributed to manuscript brushing up. All authors read and approved the final manuscript.

## Supplementary Material

Additional file 1 **Main program of the system**. Excel file including the source program for peak identification and annotation. Click here for file

Additional file 2 **Example reference library**. Excel file of an example reference library used in the main program. Click here for file

Additional file 3 **SIMCA model book**. Excel file for SIMCA method used in the main program. Click here for file

Additional file 4 **Manual**. The manual for using our system. Click here for file

Additional file 5 **Example raw data**. Example of a raw data file in standard mixture experiment. Click here for file

Additional file 6 **Example CSV file**. Example of a CSV file from MetAlign. Click here for file

Additional file 7 **Example peak table**. Example of the peak table exported from the system. Click here for file
